# Chromosome 14q loss is associated with adverse survival and a distinct transcriptional phenotype in IDH-wildtype glioblastoma

**DOI:** 10.1007/s11060-026-05855-2

**Published:** 2026-11-10

**Authors:** Nayuta Higa, Seiya Yokoyama, Toshiaki Akahane, Taiji Hamada, Hajime Yonezawa, Ryutaro Makino, Tomoko Takajo, Kei Matsuo, Mari Kirishima, Ikumi Kitazono, Yutaka Fujioka, Ryusuke Hatae, Daisuke Kuga, Shohei Nagasaka, Kohei Suzuki, Junkoh Yamamoto, Koji Yoshimoto, Akihide Tanimoto, Ryosuke Hanaya

**Affiliations:** 1 Department of Neurosurgery, Kagoshima University Graduate School of Medical and Dental Sciences, 8-35-1 Sakuragaoka, Kagoshima City, Kagoshima 890-8520 Japan; 2 Department of Pathology, Kagoshima University Graduate School of Medical and Dental Sciences, 8-35-1 Sakuragaoka, Kagoshima City, Kagoshima 890-8544 Japan; 3 Center for Human Genome and Gene Analysis, Kagoshima University Hospital, 8-35-1 Sakuragaoka, Kagoshima City, Kagoshima 890-8544 Japan; 4 Department of Neurosurgery, Graduate School of Medical Sciences, Kyushu University, 3-1-1 Maidashi, Higashi-ku, Fukuoka, 812-8582 Japan; 5 Department of Neurosurgery, University of Occupational and Environmental Health, Yahatanishi-ku, Kitakyushu, 807-8555 Japan

**Keywords:** Glioblastoma, IDH-wildtype, Chromosome 14q, Copy-number alteration, Prognostic marker, Transcriptomics, Gene set enrichment analysis, Cross-cohort heterogeneity

## Abstract

**Purpose:**

In IDH-wildtype glioblastoma, prognostic biomarkers beyond *MGMT* promoter methylation remain limited. Chromosome 14q contains candidate tumor suppressors, yet the significance of arm-level 14q loss in molecularly defined glioblastoma is unclear.

**Methods:**

Arm-level 14q status was inferred from coordinated 14q expression (Gaussian-mixture model) in bulk RNA sequencing of 215 Japanese IDH-wildtype glioblastomas and validated against methylation-array copy number (*n* = 42). Associations with outcome and transcriptional programs were evaluated and assessed in CGGA (*n* = 74) and TCGA (*n* = 139) using the discovery-cohort threshold.

**Results:**

RNA-seq-based calls agreed with array copy number in 40/42 cases (κ = 0.90). 14q loss (87/215, 40.5%) was associated with shorter overall (*p* = 0.0053) and progression-free survival (*p* = 0.0175), remaining prognostic in the prespecified multivariable model (hazard ratio 1.44, 95% confidence interval 1.02–2.01; *p* = 0.036). 14q loss and *MGMT* promoter hypomethylation were prognostically complementary (four-group *p* < 0.0001). Chromosome 14-excluded, purity-adjusted gene set enrichment analysis showed increased E2F-target and G2M-checkpoint and reduced oxidative-phosphorylation and lipid-metabolism programs in 14q-loss tumors. The survival association was reproduced in CGGA (*p* = 0.0012) but not in TCGA (*p* = 0.55), with significant heterogeneity (interaction *p* = 0.005).

**Conclusion:**

RNA-seq-inferable 14q loss identifies a clinically adverse subgroup of Japanese IDH-wildtype glioblastoma, is complementary to *MGMT* promoter methylation, and is associated with a proliferation-high, oxidative-phosphorylation-low transcriptional state. The absence of this association in TCGA underscores the need for validation in additional population-diverse cohorts before clinical implementation.

**Supplementary Information:**

The online version contains supplementary material available at https://doi.org/10.1007/s11060-026-05855-2.

## Introduction

Glioblastoma, IDH-wildtype, is the most common malignant primary brain tumor in adults and remains associated with a median overall survival (OS) of approximately 15 months despite maximal safe resection followed by temozolomide-based chemoradiotherapy [1, 2]. The 2021 World Health Organization (WHO) classification defines glioblastoma by IDH-wildtype status together with characteristic histological or molecular features [3], and current guidelines emphasize molecularly informed management [4]. Nevertheless, *MGMT* promoter methylation remains the principal molecular biomarker in routine clinical use and is particularly informative with respect to benefit from alkylating chemotherapy [5]. Biomarkers that capture additional, orthogonal tumor biology are therefore needed.

The genomic landscape of IDH-wildtype glioblastoma is dominated by combined chromosome 7 gain and chromosome 10 loss, *TERT* promoter mutation, *EGFR* amplification, and *CDKN2A/B* homozygous deletion [6]. Beyond these hallmark events, recurrent arm-level somatic copy-number alterations, including 13q and 14q deletions, were identified in early genome-wide studies of glioma [7]. High-resolution allelotyping identified 14q loss as a recurrent alteration in astrocytic tumors, including de novo glioblastoma [8]. Because arm-level aneuploidy is increasingly recognized as a functional determinant of tumor phenotype and of immune context [9, 10], a specific arm-level event such as 14q loss could in principle define a biologically and clinically distinct subgroup. Whether it does so within molecularly defined IDH-wildtype glioblastoma remains unclear.

Chromosome 14q is a compelling candidate because it harbors multiple genes with tumor-suppressive or immune-regulatory functions, including *NFKBIA* (14q13), *NDRG2* (14q11), and *MEG3*, *DICER1*, and *TRAF3* (all 14q32) [11–15]. Whereas earlier work has centered on focal *NFKBIA* deletion [11], arm-level loss would reduce the dosage of several of these loci simultaneously, potentially producing a phenotype not captured by any single-gene event.

A second challenge is generalizability: copy-number alteration profiles and their prognostic associations can differ substantially among glioblastoma cohorts [16, 17]. A candidate marker therefore requires explicit cross-cohort assessment—here in the Chinese Glioma Genome Atlas (CGGA) [18] and TCGA—rather than an assumption of uniform transferability.

Here, we inferred arm-level 14q status directly from bulk RNA sequencing in 215 consecutive Japanese IDH-wildtype glioblastomas, validated the calls against DNA-methylation-array-derived copy number, and tested the hypothesis that 14q loss is independently associated with outcome and a distinct transcriptional phenotype. We then assessed the 14q-survival association in the external CGGA and TCGA cohorts and formally tested its heterogeneity across cohort groups.

## Materials and methods

### Patients and study design

We retrospectively analyzed 215 consecutive adult patients with newly diagnosed IDH-wildtype glioblastoma, classified according to WHO 2021 criteria [3], who underwent surgical resection or biopsy between 2014 and 2024 at Kagoshima University Hospital and affiliated institutions and for whom tumor tissue of adequate quality for RNA sequencing was available. Of 324 patients identified from the surgical series, 109 had no DNA- and RNA-sequencing data, because fresh-frozen tumor tissue of adequate quantity was not available (only a biopsy had been performed in 44 of the 109) or because the extracted nucleic acids did not meet the quality requirements for library preparation; all 215 patients with both DNA- and RNA-sequencing data were analyzed, and patient selection is summarized in Supplementary Fig. 1. Patients were generally treated with maximal safe resection followed by radiotherapy with concomitant and adjuvant temozolomide; bevacizumab and tumor-treating fields were used according to Japanese clinical practice. OS was defined as the interval from initial surgery to death from any cause, and progression-free survival (PFS) as the interval from initial surgery to radiological or clinical progression or death. The study was approved by the Institutional Review Board of Kagoshima University Hospital (approval nos. 180104 and 260012) and conducted in accordance with the Declaration of Helsinki. Written informed consent was obtained from patients enrolled from 2018 onward, and an opt-out approach was used for earlier cases.

### Histological examination

Resected tumor tissues were fixed in 10% neutral buffered formalin and processed routinely as formalin-fixed, paraffin-embedded (FFPE) specimens for histological and genomic evaluation. Representative FFPE sections were reviewed histologically, and molecular classification according to the WHO 2021 criteria was performed using a bimodal DNA/RNA panel by board-certified surgical and molecular pathologists (M.K. and A.T.).

### DNA and RNA extraction

DNA and RNA were extracted from FFPE sections (10-μm thickness) and fresh-frozen tissue samples using the AllPrep DNA/RNA Mini Kit (QIAGEN, Hilden, Germany) according to the manufacturer’s instructions. Separate sequencing libraries were prepared from the DNA and RNA fractions. For DNA library preparation, DNA concentration was measured using the Qubit 3.0 Fluorometer with the dsDNA BR Assay Kit (Life Technologies, Carlsbad, CA, USA), and DNA quality was assessed using the QIAseq DNA QuantiMIZE Kit (QIAGEN). Only samples with a DNA quality check score < 0.04 were used for library preparation. DNA served as the template for polymerase chain reaction (PCR) amplification and NGS library preparation using the QIAseq Multimodal Panel (QIAGEN). For RNA library preparation, RNA was enzymatically polyadenylated at the 3′ end and reverse-transcribed into complementary DNA (cDNA). Template-switching oligonucleotides carrying universal primer sequences were incorporated at both ends of the cDNA, followed by target-enrichment PCR amplification using fusion gene-specific primers. This workflow reduces genomic DNA interference during amplification and enables the detection of fusion transcripts involving previously uncharacterized partner genes.

### NGS analysis

NGS analysis was performed using a custom DNA panel, as previously described [19, 20]. Libraries were sequenced on the NextSeq 1000 system (Illumina, San Diego, CA, USA). Amplicon sequences were aligned to the human reference genome GRCh37/hg19 within the target regions. The data were analyzed using the QIAGEN Web Portal service and reviewed with support from Mitsubishi Electric Software. Copy-number variations were calculated using the QIAGEN cloud analysis pipeline and corrected for tumor content. Amplification was defined as > 5 copies, hemizygous deletion as 0.5–1 copy, and homozygous deletion as <0.5 copies.

### Methylation-specific PCR for *MGMT* promoter

Methylation-specific PCR was performed on genomic DNA as previously reported [21]. The *MGMTp* hypomethylation index was calculated as the percentage of the intensity of the unmethylated bands to the total band intensity, as quantified using a microchip automated electrophoresis instrument (MCE-202 MultiNA; Shimadzu, Kyoto, Japan) with a DNA-1000 kit (Shimadzu). MGMT promoter methylation status was evaluable in 140 of 215 tumors.

### Bulk RNA-sequencing and comprehensive mRNA expression analysis

Bulk RNA sequencing was performed on fresh-frozen tumor specimens for transcriptome-wide expression profiling, with data available for all 215 tumors. Hematoxylin- and eosin-stained frozen sections were prepared from resected tumor tissue to confirm tumor presence; the remaining tissue was stored at − 80 °C until use. Total RNA was extracted from the fresh-frozen specimens and sequenced to a depth of approximately 12 GB of paired-end reads per sample. Raw reads were processed with Cutadapt (v5.2) to remove adapter sequences and low-quality bases, aligned to the human reference genome (hg38) using STAR, and quantified at the gene level using featureCounts with GENCODE v47 annotations. Read counts were normalized for sequencing depth and log2-transformed to generate the expression matrix used in the functional-signature analyses.

### Inference of arm-level 14q status from RNA-seq

**Fig. 1:**
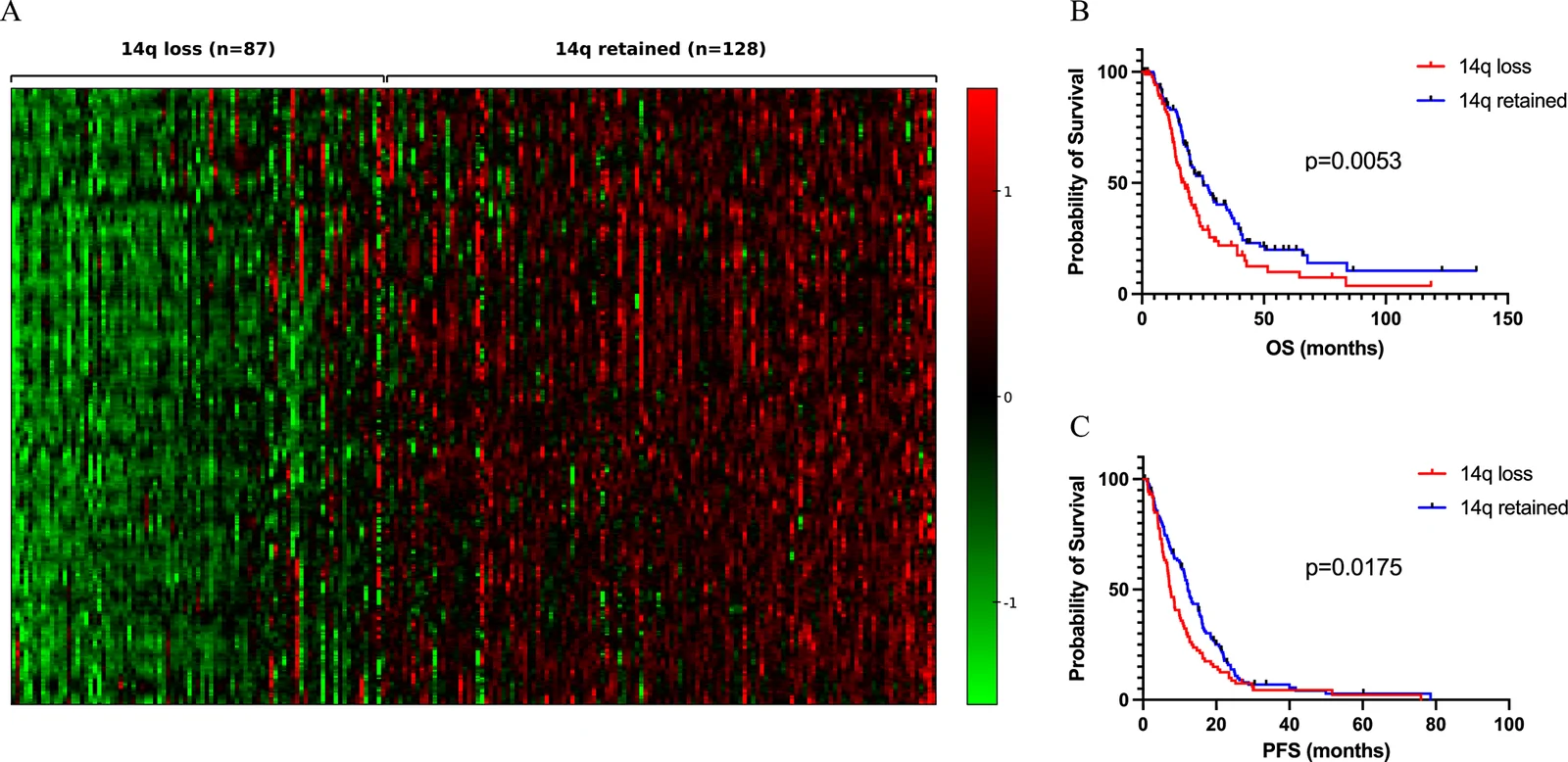
Chromosome 14q loss defined by RNA-seq and its association with survival in our cohort (*n* = 215). **A** heatmap of z-scored expression (centered within each sample) of 408 protein-coding chromosome 14q genes (rows, ordered by genomic position) across 215 IDH-wildtype glioblastomas (columns), grouped by 14q status (loss, *n* = 87; retained, *n* = 128). For display, values were averaged over a 10-gene sliding window along the genomic axis, and the color scale was truncated at ±1.5. Coordinated down-regulation across the arm is evident in the 14q-loss group. **B** Kaplan–Meier curves of overall survival (OS) by 14q status (log-rank *p* = 0.0053). C Kaplan–Meier curves of progression-free survival (PFS) by 14q status (log-rank *p* = 0.0175)

Arm-level 14q dosage was inferred from the coordinated expression of contiguous genes, an approach conceptually supported by RNA-based copy-number inference methods [22, 23]. Nuclear genes were mapped to a common genomic-coordinate reference, mitochondrial genes were excluded, and genes were retained when mean log2 expression exceeded 1.5 in the relevant sequencing batch. Expression values were median-centered per gene within each batch, each sample’s genome-wide median was subtracted as a baseline correction, and values were truncated at ±1.5 to limit the influence of focal amplifications or deletions. The chromosome 14q copy-number score was defined as the mean of these centered values across retained 14q genes. A two-component Gaussian mixture model [24] was fitted by expectation–maximization to the score distribution within each cohort, using the scikit-learn implementation with 30 random initializations and a fixed random seed (random_state = 0). Samples assigned by maximum posterior probability to the lower-mean component were classified as 14q loss; the remainder were classified as 14q retained; the threshold corresponding to a posterior probability of 0.5 was a 14q score of 0.019. To avoid a cohort-dependent definition of 14q loss, this threshold derived from our cohort was applied unchanged to the CGGA and TCGA scores, so that 14q loss was defined identically (score < 0.019) in all three cohorts. For visualization in Fig. 1A, z-scored expression of 408 protein-coding 14q genes was displayed in genomic order and smoothed for presentation with a 10-gene sliding window.

### Validation against DNA-methylation-array copy number

Genome-wide DNA methylation data were available for 42 tumors and were generated with the Infinium MethylationEPIC v2.0 BeadChip [25]. Copy-number profiles were derived from methylation-array intensities using conumee 2.0 [26]. Arm-level 14q status was assigned from the segmented conumee copy-number profiles, and the resulting categorical array call served as the reference for validation of the RNA-derived score. The continuous RNA-seq 14q score was evaluated by receiver operating characteristic (ROC) analysis and by Spearman correlation with the array-derived 14q log2 ratio, and categorical agreement was summarized by sensitivity, specificity, overall concordance, and Cohen’s κ, with exact (Clopper–Pearson) 95% CIs for proportions and bootstrap 95% CIs (5,000 resamples) for κ. Tumors with and without array data were compared to assess the representativeness of the validation subset. DNA-methylation classifier family assignments were obtained with the Heidelberg Brain Tumor Classifier (version 12.8) [27] and compared between 14q groups.

### External cohorts

For external assessment, we analyzed (i) the CGGA mRNAseq_325 dataset [18], restricted to primary IDH-wildtype glioblastoma with available survival data (*n* = 74), and (ii) the TCGA glioblastoma cohort [6] accessed through cBioPortal [28], restricted to primary, untreated de novo, non-G-CIMP glioblastomas with RNA-seq and clinical information (*n* = 139), of whom 138 had evaluable OS data. The same RNA-derived 14q scoring procedure was applied within each cohort (for CGGA, expression filtering and centering used the complete mRNAseq_325 batch as the reference population), and tumors were classified as 14q loss when the score fell below the threshold derived from our cohort of 0.019. After filtering, 419, 391, and 401 chromosome 14q genes contributed to the score in our cohort and the CGGA and TCGA datasets, respectively. For TCGA, arm-level somatic copy-number calls derived from Affymetrix SNP 6.0 arrays were obtained from the PanCanAtlas aneuploidy analysis [9], together with ABSOLUTE-estimated tumor purity from the same data release (available for 133 of 139 tumors); tumors with an arm-level call of − 1 were classified as DNA-defined 14q loss and those with a call of 0 or + 1 as retained. Calls were available for 116 of the 139 tumors. Agreement between RNA-derived and SNP-array-derived 14q status was summarized in the same way as for the methylation-array validation (ROC analysis of the continuous score and sensitivity, specificity, overall concordance, and Cohen’s κ at the discovery-cohort threshold), and OS was compared according to DNA-defined 14q status. Somatic mutations (whole-exome sequencing; 128 of 139 tumors) and GISTIC copy-number calls (135 of 139) for TCGA were obtained from cBioPortal (study gbm_tcga) and compared with the panel results using platform-comparable definitions (non-synonymous mutation; high-level amplification, GISTIC value 2; homozygous deletion of CDKN2A/B, GISTIC value −2).

### Gene set enrichment analysis

Preranked GSEA [29] was performed using the MSigDB Hallmark gene-set collection [30]. Tumor purity was estimated from bulk expression with the ESTIMATE algorithm [31]. Because bulk enrichment can reflect cellular composition, the ranking metric for each gene was the t statistic for 14q status (loss versus retained) from a linear model that additionally included ESTIMATE tumor purity. To prevent direct chromosome-dosage effects from driving enrichment, all genes located on chromosome 14 were removed before GSEA. After expression filtering (median log2 expression > 2) and removal of zero-variance genes, the final chromosome 14-excluded ranked list contained 12,067 genes. Enrichment was assessed using 1,000 gene-set permutations with a fixed random seed, and false discovery rate (FDR) q < 0.05 was considered significant.

### Statistical analysis

Continuous variables were compared with the Mann–Whitney U test and categorical variables with Fisher’s exact test or the chi-square test as appropriate; characteristics of the three cohorts were compared with the Kruskal–Wallis or chi-square test. For baseline comparisons in the discovery cohort, Benjamini–Hochberg correction was applied across tested variables [32]. Survival was estimated with the Kaplan–Meier method and compared with the log-rank test; median follow-up was estimated by the reverse Kaplan–Meier method. Cox proportional-hazards regression was used for univariable and multivariable analyses. The prespecified multivariable OS model included 14q status, age, preoperative KPS, total/subtotal resection, chemoradiotherapy, TERT promoter mutation, EGFR mutation and/or amplification, PDGFRA mutation and/or amplification, PTEN mutation and/or loss, and CDKN2A/B homozygous deletion. Sensitivity analyses added bevacizumab, tumor-treating fields, year of surgery (continuous), and the number of canonical copy-number events of glioblastoma (+7, −10, 9p loss, 13q loss, +19, and +20, called from RNA-seq dosage scores derived as for 14q) to this model. Expression of the candidate 14q genes was related to the 14q score by Spearman correlation and to OS per standard deviation of log2 expression in univariable and prespecified multivariable Cox models, with Benjamini–Hochberg correction across the five genes. Within the external cohorts, 14q status was additionally adjusted for the available covariates (age, sex, radiotherapy, temozolomide and, where available, MGMT status, and tumor purity), and heterogeneity of the 14q effect between the East Asian cohorts and TCGA was tested with a cohort-stratified Cox model containing a 14q × cohort-group interaction term, also after adjustment for age and sex. The proportional-hazards assumption for the primary 14q variable in the prespecified multivariable model was assessed using scaled Schoenfeld residuals against rank-transformed event time; no evidence of violation was detected (*p* = 0.31). All tests were two-sided, with *p* < 0.05 considered significant. Analyses were performed in Python 3.12 using pandas, SciPy, scikit-learn, lifelines, and statsmodels; preranked GSEA was performed with GSEApy [33], and the enrichment plots were generated with the Broad Institute GSEA application from the same ranked list.

## Results

### RNA-seq-derived 14q status shows high concordance with DNA-methylation-array copy number

We first evaluated the concordance of RNA-seq-derived 14q status with DNA-methylation-array copy number. Among 42 patients with paired genome-wide DNA-methylation-array data, RNA-seq-derived and array-derived copy-number profiles were visually concordant across the genome, including chromosome 14 (Supplementary Fig. 2A). The 42 tumors with array data were representative of the cohort (RNA-defined 14q loss in 40.5% vs. 40.5% of the remaining 173 tumors; median age 64.5 vs. 66 years). The continuous RNA-seq 14q score correlated with the array-derived 14q log2 ratio (Spearman ρ = 0.73; *p* < 0.001) and discriminated array-defined 14q loss with an area under the ROC curve of 0.945 (95% CI 0.842–1.000); at the classification threshold (0.019), sensitivity was 89.5% (95% CI 66.9–98.7%) and specificity was 100% (95% CI 85.2–100%) (Supplementary Fig. 2B). Categorical calls agreed in 40 of 42 cases (95.2%; Cohen’s κ = 0.90, 95% CI 0.75–1.00; Supplementary Table 1). Both discordant cases were array-loss/RNA-retained. Because the RNA score averages dosage across the arm and the two assays were performed on separate tissue aliquots, this pattern is more consistent with intratumoral heterogeneity or subclonal loss than with a systematic property of the RNA-based call. Applying the classifier to the full cohort identified 14q loss in 87 of 215 tumors (40.5%), with coordinated down-regulation of genes along the arm (Fig. 1A). DNA-methylation classifier family assignments did not differ between 14q-loss and 14q-retained tumors (glioblastoma family, 58.8% vs. 56.0%; *p* = 0.48; Supplementary Table 2), indicating that the 14q grouping did not simply recapitulate methylation-class heterogeneity.

### Clinical and molecular characteristics are broadly comparable between 14q groups

We next compared clinical and molecular characteristics between 14q-loss and 14q-retained tumors (Table 1). Age, preoperative KPS, Ki-67 labeling index, extent of resection, use of temozolomide, radiotherapy, or bevacizumab, and *MGMT* promoter methylation were comparable between groups. Among molecular alterations, *TERT* promoter mutation, *EGFR*, *PDGFRA*, *PTEN*, *NF1*, *ATRX*, *RB1*, *ATM*, and *CDKN2A/B* homozygous deletion did not differ significantly. Male sex (64.4% vs. 49.2%; *p* = 0.036) and *TP53* alteration (49.4% vs. 34.4%; *p* = 0.034) showed nominal differences, but neither survived FDR correction (q = 0.341 for both). The frequency of 14q loss did not vary with year of surgery (Spearman ρ between year and the 14q score −0.05; *p* = 0.49). The genomic landscape ordered by 14q status is shown in Supplementary Fig. 3 and did not identify a single conspicuous co-segregating driver alteration.

**Table 1:** Clinical and molecular characteristics of IDH-wildtype glioblastoma by chromosome 14q status (*n* = 215)

Variable	Level	14q loss	14q retained	P value	FDR q value
		(n = 87)	(n = 128)		
Clinical factors					
Age (years)	median [IQR]	67.0 [56.0–75.0]	64.5 [56.8–72.2]	0.186	0.551
Sex	male	56/87 (64.4%)	63/128 (49.2%)	0.036*	0.341
Preoperative KPS	median [IQR]	70.0 [60.0–90.0] (n = 86)	80.0 [60.0–90.0]	0.592	0.73
Ki-67 index (%)	median [IQR]	38.0 [25.0–58.1] (n = 86)	40.0 [27.5–50.0] (n = 123)	0.848	0.895
Extent of resection	Total/Subtotal	42/87 (48.3%)	69/128 (53.9%)	0.487	0.661
Chemotherapy	TMZ	85 (97.7%)	121 (94.5%)	0.466	0.661
	Other	1 (1.1%)	2 (1.6%)		
	No	1 (1.1%)	5 (3.9%)		
Radiotherapy	Yes	84/87 (96.6%)	121/128 (94.5%)	0.743	0.831
Bevacizumab	Yes	66/87 (75.9%)	89/128 (69.5%)	0.354	0.612
Molecular factors					
TERTp mut	mut	55/87 (63.2%)	92/128 (71.9%)	0.232	0.551
EGFR mut and/or amp	positive	27/87 (31.0%)	51/128 (39.8%)	0.197	0.551
PDGFRA mut and/or amp	positive	20/87 (23.0%)	20/128 (15.6%)	0.212	0.551
TP53 mut and/or loss	positive	43/87 (49.4%)	44/128 (34.4%)	0.034*	0.341
PTEN mut and/or loss	positive	50/87 (57.5%)	82/128 (64.1%)	0.392	0.621
CDKN2A/B homozygous deletion	homdel	44/87 (50.6%)	63/127 (49.6%)	1	1
NF1 mut and/or loss	positive	17/87 (19.5%)	30/128 (23.4%)	0.614	0.73
ATRX mut and/or loss	positive	26/87 (29.9%)	27/128 (21.1%)	0.15	0.551
RB1 mut and/or loss	positive	43/87 (49.4%)	47/128 (36.7%)	0.069	0.435
ATM mut and/or loss	positive	26/87 (29.9%)	29/128 (22.7%)	0.266	0.562
MGMT promoter methylation	Hyper-methylation	21/59 (35.6%)	36/81 (44.4%)	0.303	0.575

*Unadjusted *p* < 0.05. FDR q values were obtained by Benjamini–Hochberg correction

IQR, interquartile range; KPS, Karnofsky Performance Status; TMZ, temozolomide; mut, mutation; amp, amplification; homdel, homozygous deletion; FDR, false discovery rate

### 14q loss is associated with unfavorable survival

Patients whose tumors carried 14q loss had significantly shorter OS than those with 14q-retained tumors (median 17.5 vs. 25.1 months; log-rank *p* = 0.0053; Fig. 1B). PFS was likewise shorter in the 14q-loss group (median 7.5 vs. 12.3 months; *p* = 0.0175; Fig. 1C).

In the multivariable model, 14q loss remained associated with worse OS (HR 1.44, 95% CI 1.02–2.01; *p* = 0.036), together with total/subtotal resection (HR 0.63, 95% CI 0.45–0.89; *p* = 0.009), chemoradiotherapy (HR 0.30, 95% CI 0.14–0.62; *p* = 0.001), and *PDGFRA* alteration (HR 1.78, 95% CI 1.14–2.79; *p* = 0.012) (Table 2). The association was consistent across sensitivity analyses (hazard ratios 1.41–1.48 in the extended models of Supplementary Table 3): the addition of bevacizumab (HR 1.41, 95% CI 1.00–1.98), tumor-treating fields (HR 1.46, 95% CI 1.04–2.05), year of surgery (HR 1.48, 95% CI 1.05–2.08; *p* = 0.025), or the three together (HR 1.45, 95% CI 1.02–2.04; *p* = 0.036) left the estimate essentially unchanged.

**Table 2:** Univariate and multivariate Cox regression analyses of overall survival in IDH-wildtype glioblastoma

	Univariate		Multivariate	
Variable	HR (95% CI)	p value	HR (95% CI)	p value
14q loss	1.58 (1.14–2.19)	0.006	1.44 (1.02–2.01)	0.036
Age (per 10 years)	1.19 (1.04–1.36)	0.010	1.13 (0.99–1.30)	0.079
Preoperative KPS (per 10 points)	0.90 (0.84–0.97)	0.008	0.94 (0.86–1.02)	0.163
Total/Subtotal resection	0.61 (0.44–0.85)	0.003	0.63 (0.45–0.89)	0.009
Chemoradiotherapy	0.39 (0.20–0.78)	0.007	0.30 (0.14–0.62)	0.001
*TERT*p mut	1.09 (0.77–1.56)	0.623	1.42 (0.93–2.15)	0.103
*EGFR* mut and/or amp	0.68 (0.48–0.96)	0.026	0.75 (0.51–1.09)	0.134
*PDGFRA* mut and/or amp	2.05 (1.38–3.05)	<0.001	1.78 (1.14–2.79)	0.012
*PTEN* mut and/or loss	0.75 (0.54–1.04)	0.087	0.79 (0.54–1.14)	0.207
*CDKN2A/B* homozygous deletion	1.27 (0.92–1.76)	0.140	1.16 (0.82–1.63)	0.398

HR, hazard ratio; CI, confidence interval; KPS, Karnofsky Performance Status; mut, mutation; amp, amplification. Bold indicates *p* < 0.05

We next asked whether 14q loss tracks the canonical copy-number events of glioblastoma (+7, −10, 9p loss, 13q loss, +19, and +20), inferred from RNA-seq dosage scores in the same way as 14q. The number of canonical events was not associated with OS (HR per event 1.00, 95% CI 0.89–1.12), and its addition to the prespecified model left the 14q estimate unchanged (HR 1.44, 95% CI 1.02–2.02; *p* = 0.038; Supplementary Table 3).

At the level of individual 14q genes, expression of *NFKBIA*, *NDRG2*, *MEG3*, *DICER1*, and *TRAF3* correlated positively with the 14q score (Spearman ρ 0.19–0.48; all *p* ≤ 0.005) and was lower in 14q-loss than in 14q-retained tumors (median log2 differences −0.45 to −0.67; all *p* ≤ 0.004), consistent with a dosage effect of arm-level loss (Supplementary Table 4). Higher *MEG3* expression was associated with longer OS (HR per SD 0.76, 95% CI 0.63–0.90; *p* = 0.002; q = 0.010), an association that persisted after adjustment for the prespecified covariates (HR 0.67; *p* < 0.001). Thus, 14q loss reduces the expression of all candidate genes, and among them only *MEG3* expression carried independent prognostic information in the discovery cohort.

### 14q loss and MGMT promoter methylation provide complementary prognostic information

**Fig. 2:**
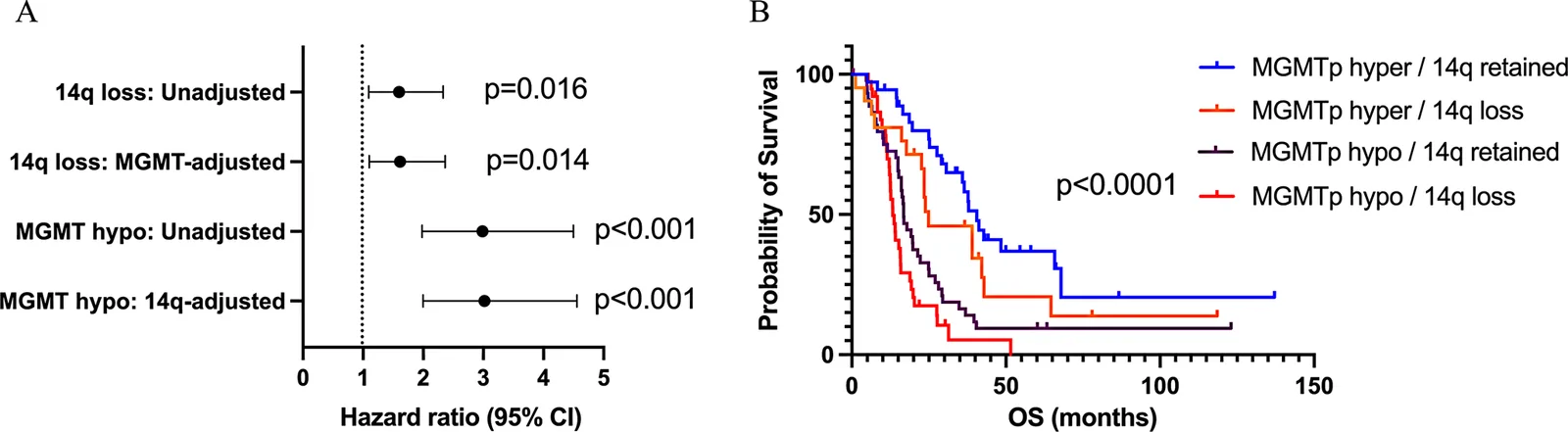
14q loss and *MGMT* promoter methylation provide complementary prognostic information (*MGMT*-evaluable subset, *n* = 140). **A** forest plot of hazard ratios (95% CI) for OS. 14q loss remained associated with OS after adjustment for *MGMT* status, and *MGMT* promoter hypomethylation remained associated with OS after adjustment for 14q status. **B** Kaplan–Meier curves of OS for the four groups defined by *MGMT* promoter status (hyper- versus hypomethylated) and 14q status (log-rank *p* < 0.0001)

Because *MGMT* promoter status was available for 140 patients, its interplay with 14q status was analyzed in this subset. 14q loss was associated with worse OS before (HR 1.60, 95% CI 1.09–2.33; *p* = 0.016) and after adjustment for *MGMT* status (HR 1.61, 95% CI 1.10–2.37; *p* = 0.014). Conversely, *MGMT* promoter hypomethylation remained associated with worse OS after adjustment for 14q status (unadjusted HR 2.99, 95% CI 1.98–4.50, *p* < 0.001; 14q-adjusted HR 3.02, 95% CI 2.00–4.56, *p* < 0.001) (Fig. 2A). Combining the two markers stratified patients into four groups with distinct OS (*p* < 0.0001; Fig. 2B): *MGMT*-hypermethylated/14q-retained tumors had the most favorable outcome, *MGMT*-hypomethylated/14q-loss tumors the least favorable, and the remaining combinations were intermediate. Thus, 14q status provides prognostic information complementary to *MGMT* promoter methylation.

### 14q loss is associated with a proliferation-high, oxidative-phosphorylation-low transcriptional state

**Fig. 3:**
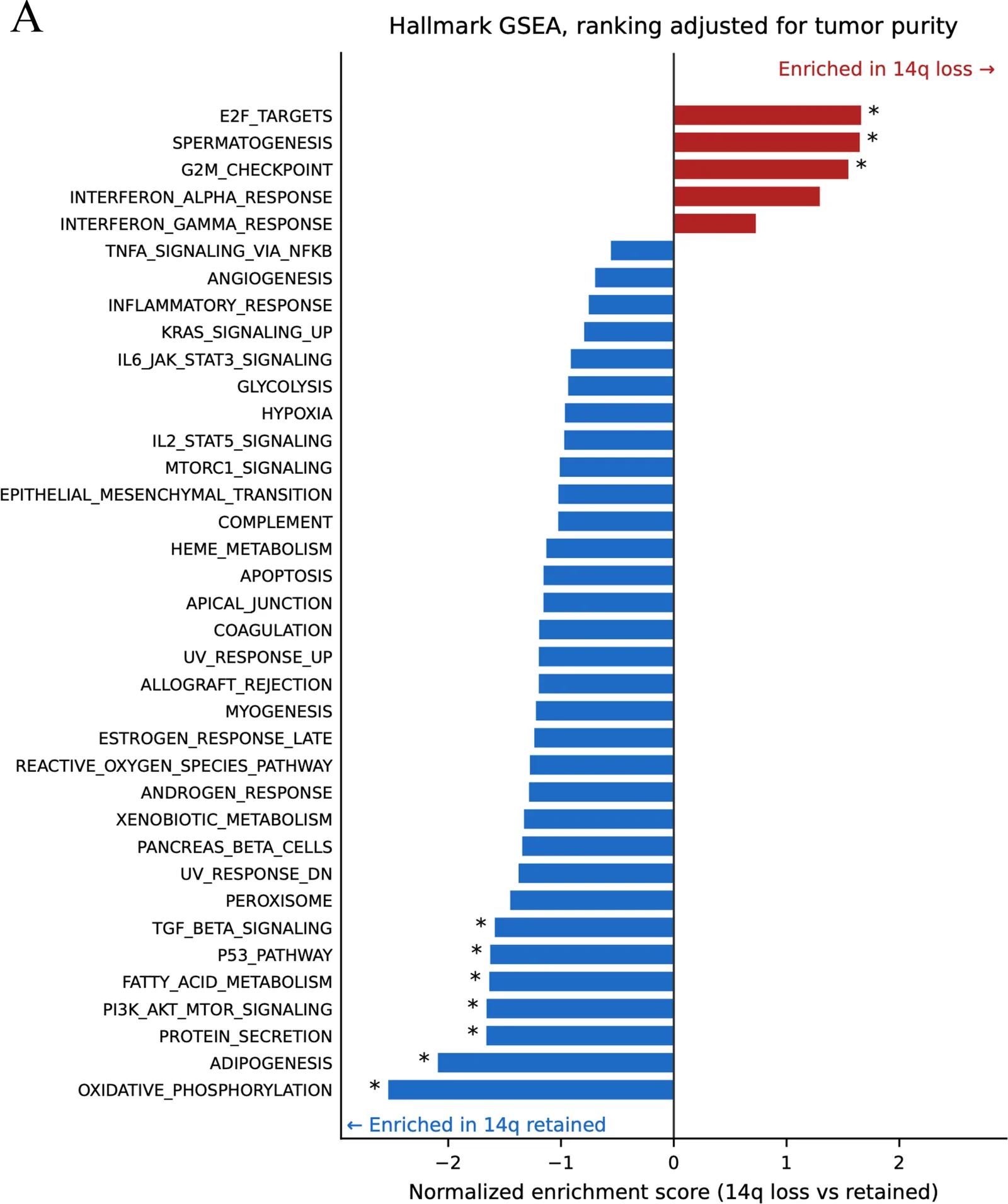

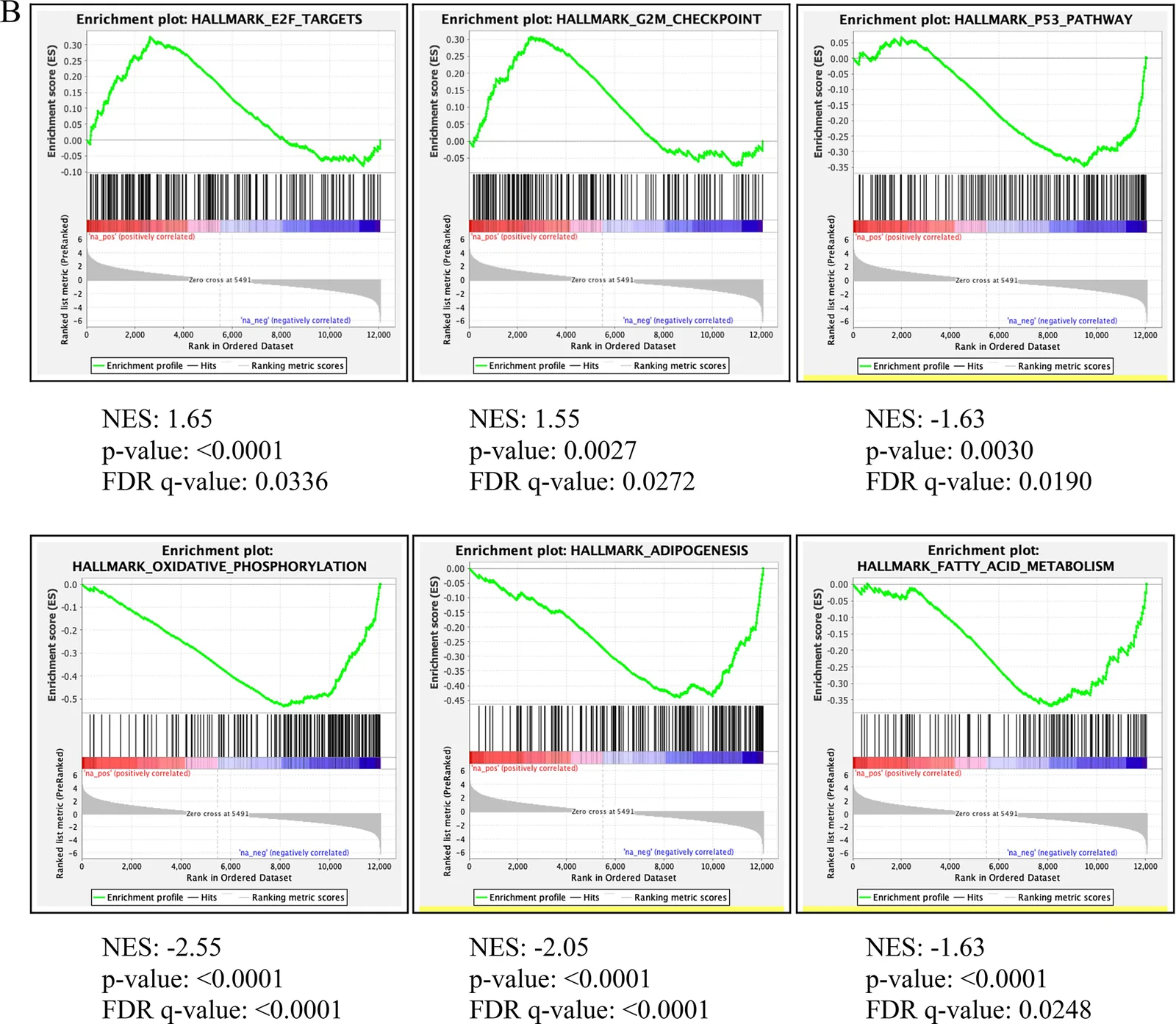
Chromosome 14-excluded Hallmark GSEA adjusted for tumor purity identifies a proliferation-high, oxidative-phosphorylation-low transcriptional pattern in 14q-loss tumors. **A** normalized enrichment scores (NES) of 37 Hallmark gene sets (14q loss versus retained; ranking metric adjusted for ESTIMATE tumor purity; all chromosome 14 genes excluded from the ranked list), ordered by NES. The panel shows the gene sets that were significant in the same analysis before adjustment for purity; of the 11 sets significant after adjustment (asterisks, FDR q < 0.05), 10 are shown and MYC targets V1 is not displayed. **B** enrichment plots on the purity-adjusted ranking generated with the GSEA application

To characterize biology associated with 14q loss while minimizing direct chromosome-dosage effects and the influence of composition, we performed preranked Hallmark GSEA after excluding all chromosome 14 genes from the ranking, with the ranking metric adjusted for tumor purity (Fig. 3A). Eleven of the 50 gene sets were significant at FDR q < 0.05, ten of which are among the 37 sets displayed in Fig. 3A. E2F targets (normalized enrichment score [NES] 1.65; q = 0.034) and G2M checkpoint (NES 1.55; q = 0.027) were enriched in 14q-loss tumors, whereas oxidative phosphorylation (NES −2.55; q < 0.001), adipogenesis (NES −2.05; q < 0.001), fatty acid metabolism (NES −1.63; q = 0.025), and the p53 pathway (NES −1.63; q = 0.019) were enriched in 14q-retained tumors (Fig. 3A, B). Because the ranked list contained no chromosome 14 genes, these signatures reflect genome-wide transcriptional programs rather than direct expression changes of genes physically located on 14q. Collectively, the data define a proliferation-high, oxidative-phosphorylation-low transcriptional state associated with 14q loss that is independent of estimated tumor purity.

### The adverse survival association is observed in CGGA but not in TCGA

With the discovery-cohort threshold applied unchanged, 14q loss was identified in 31 of 74 IDH-wildtype glioblastomas in the CGGA cohort (41.9%) and was associated with markedly shorter OS (median 10.3 vs. 19.4 months; HR 2.29, 95% CI 1.37–3.83; log-rank *p* = 0.0012; Supplementary Fig. 4A). In contrast, in TCGA, 14q loss (54/139, 38.8%) was not associated with OS (median 12.8 vs. 13.0 months; HR 0.88, 95% CI 0.59–1.31; *p* = 0.55; Supplementary Fig. 4B). The frequency of 14q loss did not differ significantly across the three cohorts (40.5%, 41.9%, and 38.8%; χ^2^*p* = 0.91) or between the pooled East Asian cohorts and TCGA (40.8% vs. 38.8%; Fisher’s exact *p* = 0.75). Baseline characteristics were balanced between 14q groups in both external cohorts (Supplementary Table 5); in CGGA, *MGMT* promoter methylation tended to be less frequent in the 14q-loss group (20.0% vs. 40.5%; *p* = 0.078), whereas radiotherapy and temozolomide use were comparable. A formal interaction test confirmed heterogeneity of the prognostic effect between the East Asian cohorts (our cohort and CGGA) and TCGA (interaction *p* = 0.005).

The three cohorts differed substantially in composition (Supplementary Table 5). Our cohort was older (median 66 vs. 54 and 62 years in CGGA and TCGA), was more uniformly treated with radiotherapy plus temozolomide (94% vs. 68% and 65%) and frequently received bevacizumab (72%; not recorded in the external datasets), and had longer median OS (21.2 vs. 13.9 and 12.8 months), whereas TCGA had the shortest follow-up (median 31 vs. 50 and 125 months). Within TCGA, no mutation, amplification, or homozygous deletion of driver genes differed significantly by 14q status (Supplementary Table 5), and the directions of the nominal differences seen in our cohort were similar in TCGA. At the cohort level, *EGFR* amplification (30% vs. 53%) and *EGFR* mutation (16% vs. 35%) were less frequent in our cohort than in TCGA. Transcriptional subtypes, assigned with the same procedure in all three cohorts, were similarly distributed (Supplementary Table 5). Within TCGA, 14q loss remained unassociated with OS after adjustment for age, sex, radiotherapy, temozolomide, and ABSOLUTE purity (HR 0.95, 95% CI 0.62–1.46), whereas in CGGA the association persisted after adjustment for age, sex, radiotherapy, temozolomide, *MGMT* status, and purity (HR 1.94, 95% CI 1.07–3.49; *p* = 0.028).

RNA-derived 14q status in TCGA (discovery-cohort threshold) agreed with SNP-array arm-level calls in 110 of 116 evaluable tumors (94.8%; Cohen’s κ = 0.88, 95% CI 0.78–0.96; AUC 0.976, 95% CI 0.927–1.000; sensitivity 97.2%, specificity 93.8%; Supplementary Fig. 5A). DNA-defined 14q loss (36/116, 31.0%) was likewise not associated with OS (median 12.8 vs. 12.7 months; HR 0.99, 95% CI 0.63–1.56; log-rank *p* = 0.98; Supplementary Fig. 5B), indicating that the non-replication in TCGA is not attributable to RNA-based misclassification.

## Discussion

This study identifies arm-level chromosome 14q loss as an RNA-seq-inferable candidate prognostic marker in IDH-wildtype glioblastoma and supports four main conclusions. First, RNA-derived 14q calls showed high agreement with methylation-array copy number in the discovery cohort and with SNP-array copy number in TCGA. Second, 14q loss was associated with shorter survival after multivariable adjustment, provided prognostic information complementary to *MGMT* promoter methylation, and was unchanged after adjustment for year of surgery, bevacizumab exposure, and the canonical copy-number events of glioblastoma. Third, after chromosome 14 genes were removed from the expression ranking and the ranking was adjusted for tumor purity, 14q-loss tumors retained a coherent proliferation-high, oxidative-phosphorylation-low transcriptional pattern. Fourth, the adverse survival association was observed in the Japanese discovery cohort and also observed in the independent Chinese CGGA cohort but not in TCGA, and a significant interaction formally demonstrated cross-cohort heterogeneity. These findings support biological and prognostic relevance for 14q loss in the East Asian cohorts while demonstrating that the prognostic association is cohort-dependent and not yet generalizable.

Recurrent 14q loss has been recognized in astrocytic tumors for over two decades [7, 8]; our study extends this literature by focusing on the arm-level event in a WHO 2021-defined IDH-wildtype glioblastoma cohort, validating an RNA-based call against methylation-array- and SNP-array-derived copy number, and examining the survival association in external cohorts. Importantly, *EGFR* alterations were not depleted in the 14q-loss group (31.0% vs. 39.8%), suggesting that arm-level 14q loss is unlikely to be explained solely by the previously described relationship between focal *NFKBIA* deletion and *EGFR* amplification [11]. Arm-level loss may instead alter the dosage of multiple genes distributed across 14q. Because the number of canonical copy-number events of glioblastoma was not prognostic and its inclusion did not alter the 14q estimate, the prognostic information carried by 14q loss does not appear to reflect these canonical events; nevertheless, we cannot exclude that 14q loss is a component of a broader pattern of recurrent arm-level losses rather than an independent biological event.

The proliferation-associated component of the phenotype has plausible mechanistic underpinnings, although the present data do not establish causality. What the present data show is that expression of *NFKBIA*, *NDRG2*, *MEG3*, *DICER1*, and *TRAF3* decreased with the 14q score, consistent with a dosage effect of arm-level loss, and that only MEG3 expression was independently associated with OS. The data therefore support a dosage effect and single out *MEG3* as the candidate most worth pursuing, but a single-cohort association does not establish mediation, and the following mechanisms remain hypotheses. Hemizygous reduction of *NDRG2* (14q11), an inhibitor of glioblastoma cell proliferation [12], together with reduced dosage of the imprinted tumor-suppressive long non-coding RNA *MEG3* (14q32) [13] and of *DICER1* (14q32), a central component of microRNA biogenesis and a haploinsufficient tumor suppressor [14], could contribute to broader de-repression of cell-cycle programs. This hypothesis is consistent with enrichment of E2F-target and G2M-checkpoint gene sets and with proliferative states described across glioblastoma subtypes and cellular programs [34, 35], and the independent prognostic association of *MEG3* expression is consistent with it, although it requires validation in independent cohorts. Notably, the Ki-67 labeling index did not differ between 14q groups, although this index is a semiquantitative estimate obtained from a single tissue section; the GSEA signal may therefore reflect a coordinated transcriptional program rather than a difference in the fraction of cycling cells. After adjustment for tumor purity, 14q-loss tumors also showed relative depletion of oxidative-phosphorylation, fatty-acid-metabolism, adipogenesis, and p53-pathway programs. The combination of a proliferation-high and mitochondrial-metabolism-low transcriptional state is reminiscent of the proliferative/progenitor rather than the mitochondrial subtype in pathway-based classifications of glioblastoma, in which the mitochondrial subtype has the most favorable outcome [36].

From a clinical standpoint, the complementarity with *MGMT* promoter methylation may be relevant for prognostic risk stratification. *MGMT* silencing is associated with benefit from alkylating chemotherapy [5], and *MGMT* status is an established prognostic and predictive factor in glioblastoma [21], yet substantial outcome heterogeneity remains within *MGMT*-defined groups. In our *MGMT*-evaluable subset, mutual adjustment left the effects of both markers essentially unchanged, and their combination separated patients into four survival groups. This complementarity is prognostic, not predictive: because nearly all patients received temozolomide and treatment at progression was not uniformly recorded, the present data do not test whether 14q status modifies the benefit of temozolomide or of any other treatment, and 14q loss should not be regarded as a predictive marker on the basis of this study.

A key finding of the external assessment is the significant heterogeneity of the survival association across cohorts, rather than the negative TCGA result alone. Prior glioblastoma studies have shown that the distribution of prognostic copy-number profiles can differ between Japanese and TCGA cohorts [16] and that selection of different glioblastoma populations can materially alter copy-number subtype frequencies [17]. We therefore examined measurable differences systematically. The cohorts differed in age, treatment intensity, bevacizumab exposure, and follow-up, but none of these accounted for the discrepancy: the TCGA estimate remained null after adjustment for the available covariates, including DNA-based purity, whereas the CGGA association persisted after adjustment, and the interaction estimate was essentially unchanged after adjustment for age and sex. Taken together, these observations indicate that the prognostic association is not universal. The prognostic value of 14q loss is at present supported only in the two East Asian cohorts, and 14q loss should be regarded as a cohort-dependent candidate marker until it has been tested in additional population-diverse cohorts.

This study has several limitations. It is retrospective, and the discovery cohort derives from a single region of Japan, although consecutive accrual and relatively uniform treatment reduce some sources of selection bias; patients without adequate fresh-frozen tissue or nucleic acids of adequate quality—most often those who underwent biopsy only—were not profiled, so that the cohort under-represents biopsy-only patients (12.1% of analyzed vs. 40.4% of non-profiled patients), although profiled and non-profiled patients had similar age, performance status, and OS (median 21.2 vs. 20.2 months; log-rank *p* = 0.83); and treatment at progression (re-resection, re-irradiation, or second-line systemic therapy) was not uniformly recorded and could not be modeled, and because almost all patients received temozolomide, interactions between 14q status and treatment could not be examined. RNA-seq is a surrogate rather than a direct measurement of DNA copy number. Although agreement with methylation-array and SNP-array copy number was high, the primary orthogonal validation set comprised only 42 tumors (19 with array-defined loss), so that the confidence intervals for sensitivity and κ were wide, and the larger TCGA validation set differed in platform, population, and analytical pipeline.

## Conclusions

Arm-level chromosome 14q loss can be inferred from bulk RNA sequencing with high agreement with methylation-array copy number. In the Japanese discovery cohort, 14q loss provides prognostic information complementary to *MGMT* promoter methylation and is associated with a proliferation-high, oxidative-phosphorylation-low transcriptional state that is independent of tumor purity. 14q loss identified a subgroup with worse survival after multivariable adjustment. The association was reproduced in CGGA but not in TCGA, with a significant cohort interaction that was not explained by measurable differences in age, treatment, or follow-up; 14q loss is therefore a cohort-dependent candidate prognostic marker rather than a marker ready for clinical implementation. Population-diverse prospective validation and a standardized calling strategy are necessary before clinical implementation.

## Electronic supplementary material

Below is the link to the electronic supplementary material.

Supplementary material 1

Supplementary material 2

Supplementary material 3

Supplementary material 4

Supplementary material 5

Supplementary material 6

Supplementary material 7

Supplementary material 8

Supplementary material 9

Supplementary material 10

## Data Availability

The datasets generated and/or analyzed during the current study are not publicly available because of ethical restrictions related to human-subject research. Access to sequencing and gene-expression data is restricted by the institutional ethics committee but may be available from the corresponding author on reasonable request and with approval from the relevant ethics committee. The data are stored in a controlled-access repository at the Department of Neurosurgery, Kagoshima University Graduate School of Medical and Dental Sciences.

## Abbreviations

AUC: Area under the curve
CGGA: Chinese Glioma Genome Atlas
CI: Confidence interval
FDR: False discovery rate
GSEA: Gene set enrichment analysis
HR: Hazard ratio
IDH: Isocitrate dehydrogenase
IQR: Interquartile range
KPS: Karnofsky Performance Status
NES: Normalized enrichment score
NGS: Next-generation sequencing
OS: Overall survival
PFS: Progression-free survival
ROC: Receiver operating characteristic
SD: Standard deviation
SNP: Single-nucleotide polymorphism
TCGA: The Cancer Genome Atlas
TMZ: Temozolomide
TTF: Tumor-treating fields
WHO: World Health Organization
