# Supplementary material for: Chromosome 14q loss is associated with adverse survival and a distinct transcriptional phenotype in IDH-wildtype glioblastoma
Source: J Neurooncol. 2026 Nov 10;180(2):43. doi: 10.1007/s11060-026-05855-2 (PMC13751740; doi:10.1007/s11060-026-05855-2)
Supplement: Supplementary file 1 [file 11060_2026_5855_MOESM1_ESM.pdf]

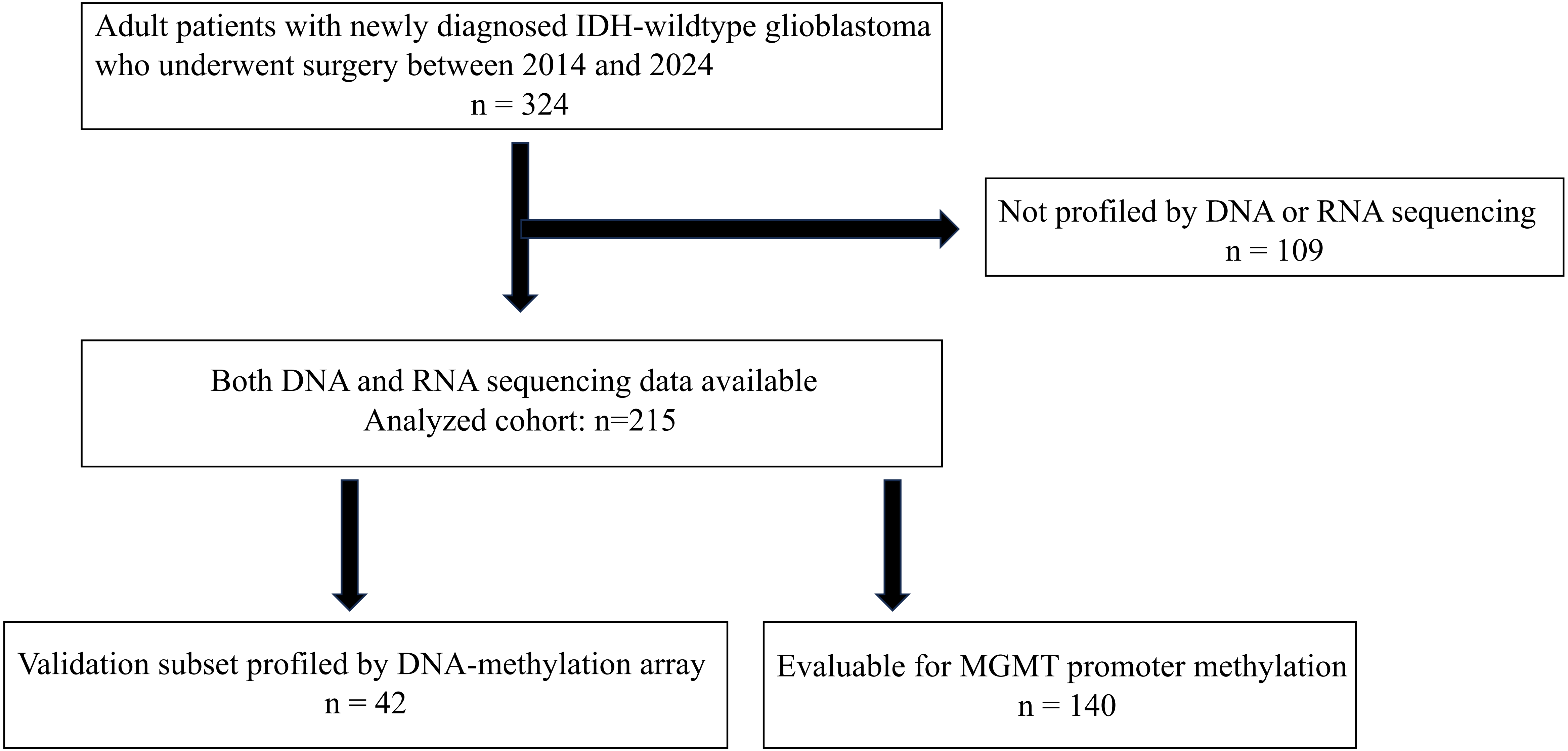

Supplementary Fig. 1 Patient selection.

Of 324 adult patients with newly diagnosed IDH-wildtype glioblastoma (WHO 2021) who underwent surgery at the participating institutions between 2014 and 2024, 109 were not profiled by DNA or RNA sequencing; the 215 patients with both DNA- and RNA-sequencing data constitute the analyzed cohort. The subsets profiled by DNA-methylation array (n = 42) and evaluable for *MGMT* promoter methylation (n = 140) are indicated.
