# Supplementary material for: Chromosome 14q loss is associated with adverse survival and a distinct transcriptional phenotype in IDH-wildtype glioblastoma
Source: J Neurooncol. 2026 Nov 10;180(2):43. doi: 10.1007/s11060-026-05855-2 (PMC13751740; doi:10.1007/s11060-026-05855-2)
Supplement: Supplementary file 2 [file 11060_2026_5855_MOESM2_ESM.pdf]

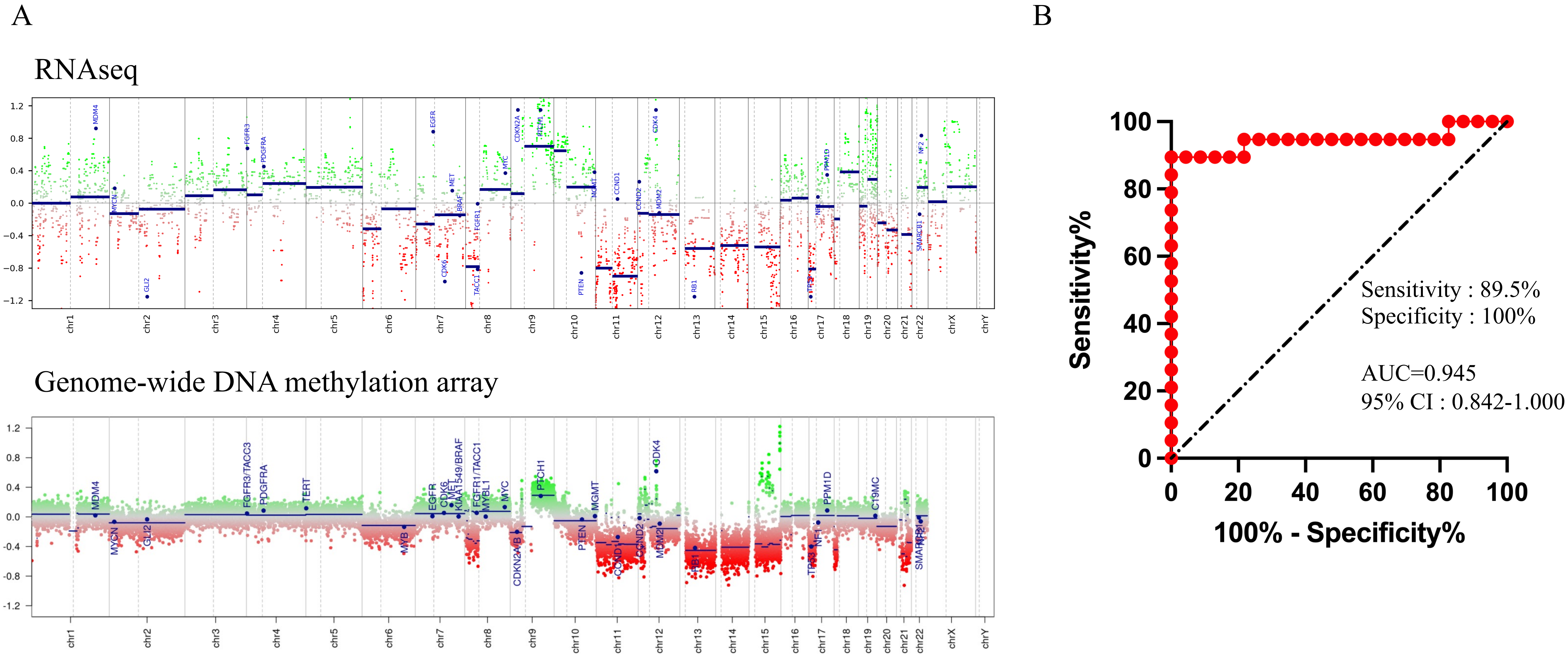

Supplementary Fig. 2 Validation of RNA-seq-based 14q calling against DNA-methylation-array copy number.

A Representative genome-wide copy-number profiles derived from RNA-seq (top) and from the EPICv2 methylation array with conumee 2.0 (bottom) in the same patient. **B** ROC curve of the continuous RNA-seq 14q score against array-defined 14q status (n = 42): AUC 0.945 (95% CI 0.842–1.000); at the classification threshold (14q score < 0.019), sensitivity was 89.5% and specificity was 100%.
