# Supplementary material for: Chromosome 14q loss is associated with adverse survival and a distinct transcriptional phenotype in IDH-wildtype glioblastoma
Source: J Neurooncol. 2026 Nov 10;180(2):43. doi: 10.1007/s11060-026-05855-2 (PMC13751740; doi:10.1007/s11060-026-05855-2)
Supplement: Supplementary file 3 [file 11060_2026_5855_MOESM3_ESM.pdf]

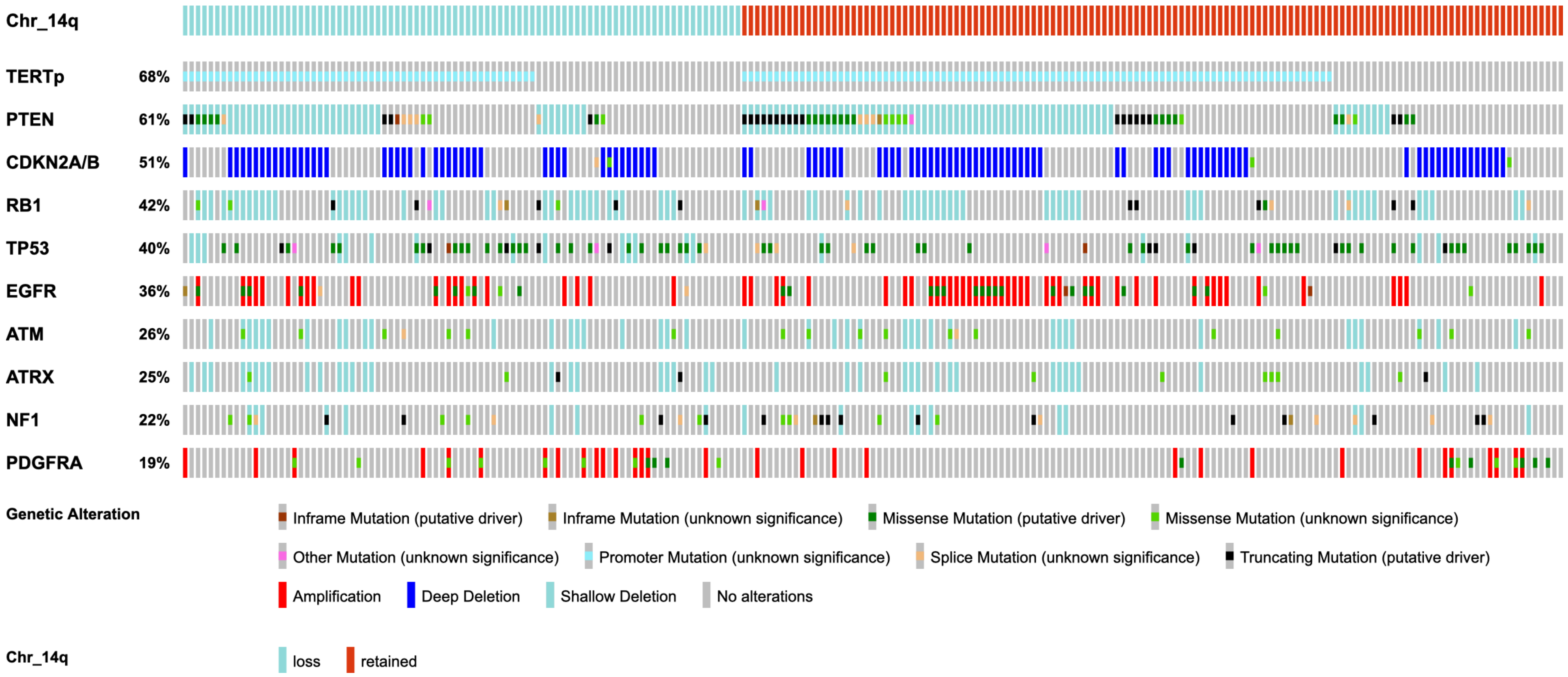

Supplementary Fig. 3 Genomic landscape by 14q status.

Oncoprint of driver alterations (*TERT* promoter, *PTEN*, *CDKN2A/B*, *RB1*, *TP53*, *EGFR*, *ATM*, *ATRX*, *NF1*, *PDGFRA*) across 215 IDH-wildtype glioblastomas, ordered by 14q status as indicated by the Chr 14q clinical track. Percentages denote overall alteration frequencies; colors denote alteration types as indicated.
