# Supplementary material for: Chromosome 14q loss is associated with adverse survival and a distinct transcriptional phenotype in IDH-wildtype glioblastoma
Source: J Neurooncol. 2026 Nov 10;180(2):43. doi: 10.1007/s11060-026-05855-2 (PMC13751740; doi:10.1007/s11060-026-05855-2)
Supplement: Supplementary file 4 [file 11060_2026_5855_MOESM4_ESM.pdf]

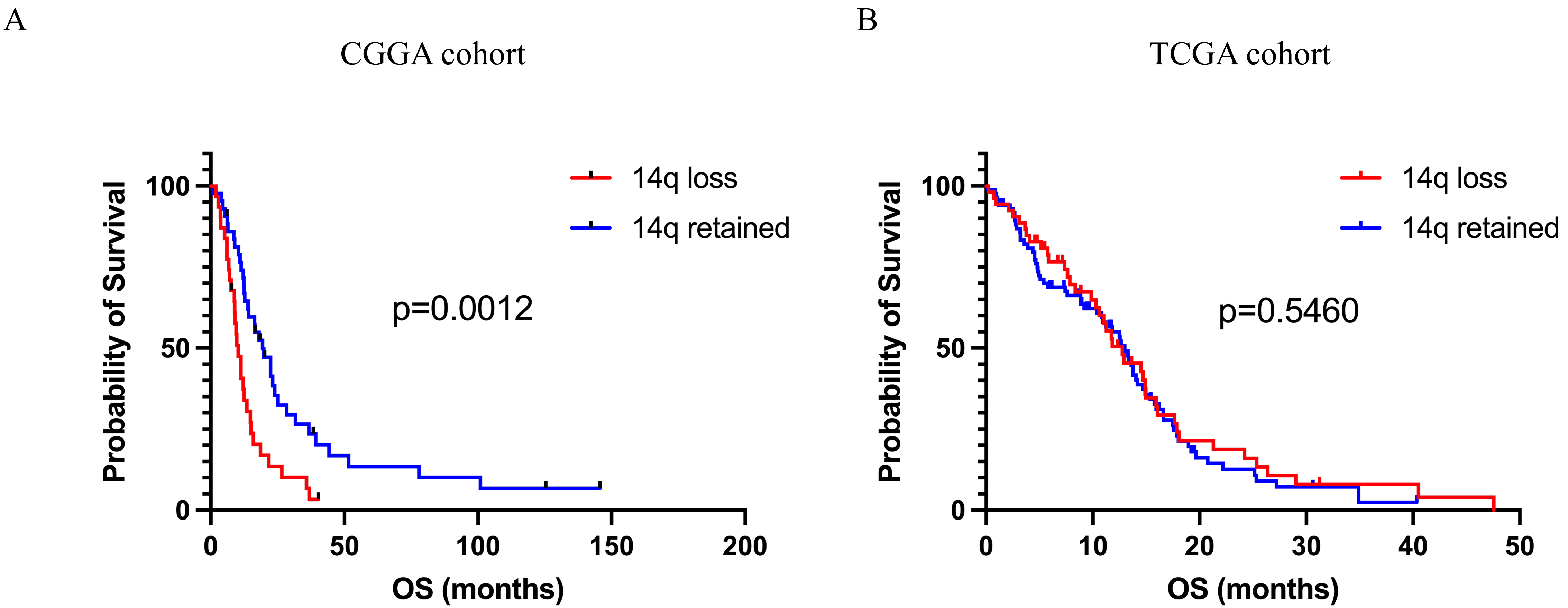

Supplementary Fig. 4 External assessment of the survival association of 14q loss.

Kaplan–Meier curves of OS by 14q status, defined by applying the threshold derived from our cohort (14q score < 0.019) unchanged, in **A** the CGGA cohort (n = 74; 14q loss, n = 31; log-rank p = 0.0012) and **B** the TCGA cohort (n = 138 with evaluable survival; 14q loss, n = 53; log-rank p = 0.55). The cohort-by-14q interaction was significant (p = 0.005), indicating heterogeneity of the survival association across cohort groups.
