# Supplementary material for: Chromosome 14q loss is associated with adverse survival and a distinct transcriptional phenotype in IDH-wildtype glioblastoma
Source: J Neurooncol. 2026 Nov 10;180(2):43. doi: 10.1007/s11060-026-05855-2 (PMC13751740; doi:10.1007/s11060-026-05855-2)
Supplement: Supplementary file 5 [file 11060_2026_5855_MOESM5_ESM.pdf]

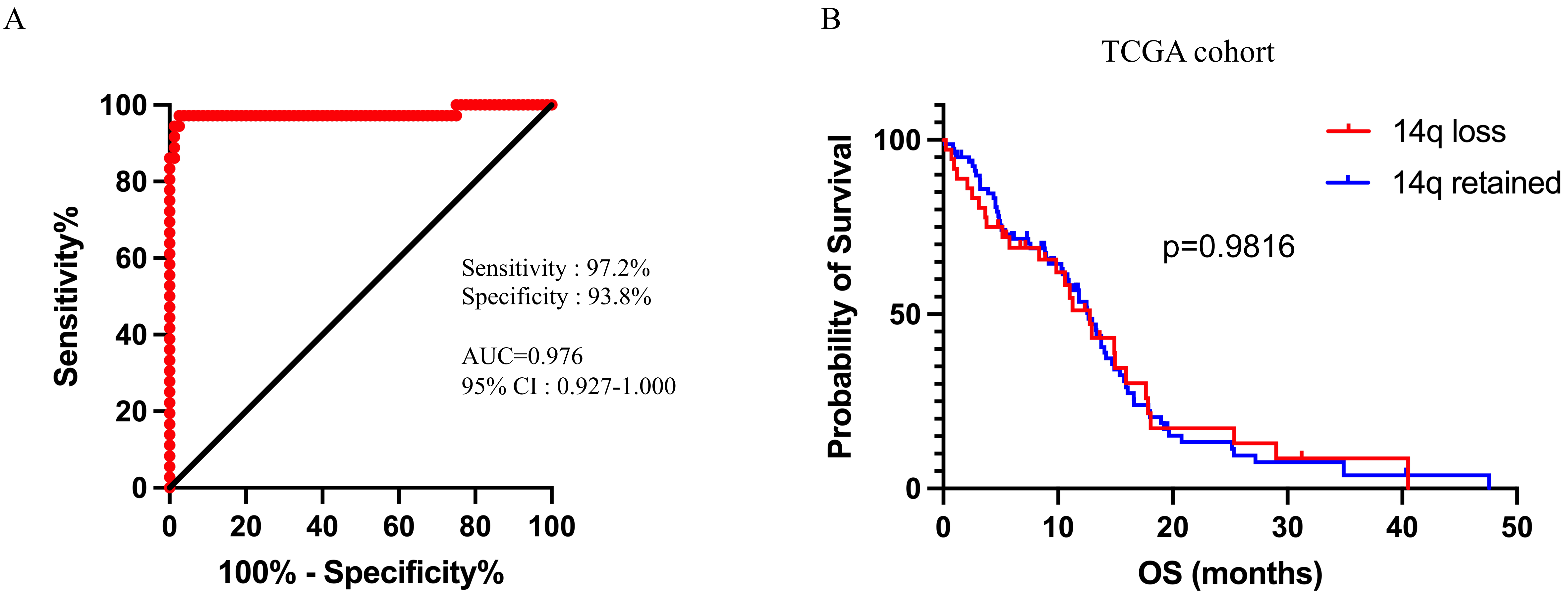

Supplementary Fig. 5 Validation of RNA-seq-based 14q calling against SNP-array copy number and survival by DNA-defined 14q status in TCGA.

A ROC curve of the continuous RNA-seq 14q score against SNP-array-derived arm-level 14q loss (PanCanAtlas arm-level calls; n = 116 tumors with evaluable calls): AUC 0.976 (95% CI 0.927–1.000); at the threshold derived from our cohort (14q score < 0.019), sensitivity was 97.2% (35/36) and specificity 93.8% (75/80) (overall concordance 110/116, 94.8%; Cohen's κ = 0.88). **B** Kaplan–Meier curves of OS according to SNP-array-defined 14q status (log-rank p = 0.9816).
