# Supplementary material for: Chromosome 14q loss is associated with adverse survival and a distinct transcriptional phenotype in IDH-wildtype glioblastoma
Source: J Neurooncol. 2026 Nov 10;180(2):43. doi: 10.1007/s11060-026-05855-2 (PMC13751740; doi:10.1007/s11060-026-05855-2)
Supplement: Supplementary file 6 [file 11060_2026_5855_MOESM10_ESM.docx]

**Supplementary Table 5. Clinical and molecular characteristics of our cohort and the CGGA and TCGA cohorts, overall and by chromosome 14q status**

| **Characteristic** | **Our cohort (n = 215)** | | | | **CGGA (n = 74)** | | | | **TCGA (n = 139)** | | | | **p across cohorts** |
| --- | --- | --- | --- | --- | --- | --- | --- | --- | --- | --- | --- | --- | --- |
|  | **All** | **14q loss** | **14q retained** | **p** | **All** | **14q loss** | **14q retained** | **p** | **All** | **14q loss** | **14q retained** | **p** |  |
| n | 215 | 87 | 128 |  | 74 | 31 | 43 |  | 139 | 54 | 85 |  |  |
| Age, years, median [IQR] | 66 [56–74] | 67 [56–75] | 64 [57–72] | 0.186 | 54 [44–59] | 57 [46–60] | 53 [43–56] | 0.170 | 62 [54–70] | 61 [53–67] | 63 [54–72] | 0.177 | <0.001 |
| Age ≥ 65 years | 116 (54.0%) | 52 (59.8%) | 64 (50.0%) | 0.166 | 4 (5.4%) | 3 (9.7%) | 1 (2.3%) | 0.303 | 56 (40.3%) | 16 (29.6%) | 40 (47.1%) | 0.051 | <0.001 |
| Male sex | 119 (55.3%) | 56 (64.4%) | 63 (49.2%) | 0.036 | 48 (64.9%) | 19 (61.3%) | 29 (67.4%) | 0.628 | 90 (64.7%) | 32 (59.3%) | 58 (68.2%) | 0.363 | 0.137 |
| Preoperative KPS, median [IQR] | 80 [60–90] | 70 [60–90] | 80 [60–90] | 0.592 | NA | NA | NA |  | 80 [60–80] | 80 [80–80] | 80 [60–80] | 0.043 | 0.945 |
| Radiotherapy | 205 (95.3%) | 84 (96.6%) | 121 (94.5%) | 0.743 | 63/71 (88.7%) | 28/30 (93.3%) | 35/41 (85.4%) | 0.453 | 106/133 (79.7%) | 44/51 (86.3%) | 62/82 (75.6%) | 0.184 | <0.001 |
| Temozolomide | 206 (95.8%) | 85 (97.7%) | 121 (94.5%) | 0.318 | 53/71 (74.6%) | 21/30 (70.0%) | 32/41 (78.0%) | 0.582 | 89/133 (66.9%) | 36/51 (70.6%) | 53/82 (64.6%) | 0.571 | <0.001 |
| Radiotherapy + temozolomide | 202 (94.0%) | 83 (95.4%) | 119 (93.0%) | 0.568 | 48/71 (67.6%) | 20/30 (66.7%) | 28/41 (68.3%) | 1.000 | 87/133 (65.4%) | 36/51 (70.6%) | 51/82 (62.2%) | 0.354 | <0.001 |
| Bevacizumab | 155 (72.1%) | 66 (75.9%) | 89 (69.5%) | 0.354 | NA | NA | NA |  | NA | NA | NA |  |  |
| *MGMT* promoter methylated | 57/140 (40.7%) | 21/59 (35.6%) | 36/81 (44.4%) | 0.303 | 23/72 (31.9%) | 6/30 (20.0%) | 17/42 (40.5%) | 0.078 | NA | NA | NA |  | 0.272 |
| Deaths | 150/215 (69.8%) | 65/87 (74.7%) | 85/128 (66.4%) |  | 64/74 (86.5%) | 29/31 (93.5%) | 35/43 (81.4%) |  | 109/138 (79.0%) | 41/53 (77.4%) | 68/85 (80.0%) |  |  |
| Median OS, months (95% CI) | 21.2 (18.9–24.9) | 17.5 (14.0–21.2) | 25.1 (19.8–30.6) | 0.005 | 13.9 (11.3–18.6) | 10.3 (7.7–13.5) | 19.4 (12.7–25.2) | 0.001 | 12.8 (10.9–14.2) | 12.8 (9.9–14.9) | 13.0 (10.4–14.2) | 0.546 | <0.001 |
| Median follow-up, months | 49.9 | 41.1 | 51.1 |  | 125.4 | 40.3 | 125.4 |  | 31.2 | 31.2 | 30.6 |  |  |
| **Driver genes** |  |  |  |  |  |  |  |  |  |  |  |  |  |
| *TERT* promoter mutation | 147 (68.4%) | 55 (63.2%) | 92 (71.9%) | 0.232 | NA | NA | NA |  | NA | NA | NA |  |  |
| *EGFR* mutation | 34 (15.8%) | 13 (14.9%) | 21 (16.4%) | 0.850 | NA | NA | NA |  | 45/128 (35.2%) | 14/48 (29.2%) | 31/80 (38.8%) | 0.340 | <0.001 |
| *EGFR* amplification | 65 (30.2%) | 21 (24.1%) | 44 (34.4%) | 0.131 | NA | NA | NA |  | 71/135 (52.6%) | 23/52 (44.2%) | 48/83 (57.8%) | 0.157 | <0.001 |
| *PDGFRA* mutation | 20 (9.3%) | 11 (12.6%) | 9 (7.0%) | 0.231 | NA | NA | NA |  | 6/128 (4.7%) | 2/48 (4.2%) | 4/80 (5.0%) | 1.000 | 0.177 |
| *PDGFRA* amplification | 31 (14.4%) | 16 (18.4%) | 15 (11.7%) | 0.235 | NA | NA | NA |  | 21/135 (15.6%) | 12/52 (23.1%) | 9/83 (10.8%) | 0.086 | 0.891 |
| *PTEN* mutation | 65 (30.2%) | 19 (21.8%) | 46 (35.9%) | 0.034 | NA | NA | NA |  | 43/128 (33.6%) | 14/48 (29.2%) | 29/80 (36.2%) | 0.445 | 0.597 |
| *CDKN2A/B* homozygous deletion | 107/214 (50.0%) | 44 (50.6%) | 63/127 (49.6%) | 1.000 | NA | NA | NA |  | 76/135 (56.3%) | 33/52 (63.5%) | 43/83 (51.8%) | 0.214 | 0.300 |
| *TP53* mutation | 76 (35.3%) | 34 (39.1%) | 42 (32.8%) | 0.384 | NA | NA | NA |  | 34/128 (26.6%) | 15/48 (31.2%) | 19/80 (23.8%) | 0.410 | 0.117 |
| *NF1* mutation | 36 (16.7%) | 13 (14.9%) | 23 (18.0%) | 0.583 | NA | NA | NA |  | 9/128 (7.0%) | 1/48 (2.1%) | 8/80 (10.0%) | 0.152 | 0.016 |
| *RB1* mutation | 23 (10.7%) | 11 (12.6%) | 12 (9.4%) | 0.503 | NA | NA | NA |  | 11/128 (8.6%) | 3/48 (6.2%) | 8/80 (10.0%) | 0.535 | 0.657 |
| *ATRX* mutation | 12 (5.6%) | 4 (4.6%) | 8 (6.2%) | 0.766 | NA | NA | NA |  | 1/128 (0.8%) | 0/48 (0.0%) | 1/80 (1.2%) | 1.000 | 0.050 |
| *ATM* mutation | 19 (8.8%) | 7 (8.0%) | 12 (9.4%) | 0.811 | NA | NA | NA |  | 2/128 (1.6%) | 0/48 (0.0%) | 2/80 (2.5%) | 0.528 | 0.013 |
| CDK4 amplification | 40/214 (18.7%) | 14 (16.1%) | 26/127 (20.5%) | 0.478 | NA | NA | NA |  | 24/135 (17.8%) | 10/52 (19.2%) | 14/83 (16.9%) | 0.818 | 0.942 |
| MDM2 amplification | 16/214 (7.5%) | 4 (4.6%) | 12/127 (9.4%) | 0.290 | NA | NA | NA |  | 11/135 (8.1%) | 3/52 (5.8%) | 8/83 (9.6%) | 0.530 | 0.982 |
| **Transcriptional subtype** |  |  |  |  |  |  |  |  |  |  |  |  |  |
| Classical | 45 (20.9%) | 12 (13.8%) | 33 (25.8%) | 0.006 | 15 (20.3%) | 3 (9.7%) | 12 (27.9%) | 0.076 | 39 (28.1%) | 9 (16.7%) | 30 (35.3%) | 0.128 | 0.723 |
| Mesenchymal | 70 (32.6%) | 25 (28.7%) | 45 (35.2%) |  | 27 (36.5%) | 16 (51.6%) | 11 (25.6%) |  | 42 (30.2%) | 19 (35.2%) | 23 (27.1%) |  |  |
| Neural | 34 (15.8%) | 12 (13.8%) | 22 (17.2%) |  | 10 (13.5%) | 3 (9.7%) | 7 (16.3%) |  | 22 (15.8%) | 10 (18.5%) | 12 (14.1%) |  |  |
| Proneural | 66 (30.7%) | 38 (43.7%) | 28 (21.9%) |  | 22 (29.7%) | 9 (29.0%) | 13 (30.2%) |  | 36 (25.9%) | 16 (29.6%) | 20 (23.5%) |  |  |

NA, not available.
