# Supplementary material for: Chromosome 14q loss is associated with adverse survival and a distinct transcriptional phenotype in IDH-wildtype glioblastoma
Source: J Neurooncol. 2026 Nov 10;180(2):43. doi: 10.1007/s11060-026-05855-2 (PMC13751740; doi:10.1007/s11060-026-05855-2)
Supplement: Supplementary file 7 [file 11060_2026_5855_MOESM6_ESM.docx]

**Supplementary Table 1.** Cross-tabulation of 14q status determined by RNA-seq and by DNA methylation array copy-number profiling (n = 42)

|  | **Methylation array 14q status** | | **Total** |
| --- | --- | --- | --- |
| **RNA-seq 14q call** | **Loss** | **Retained** |  |
| Loss | 17 | 0 | 17 |
| Retained | 2 | 23 | 25 |
| Total | 19 | 23 | 42 |
