# Supplementary material for: Chromosome 14q loss is associated with adverse survival and a distinct transcriptional phenotype in IDH-wildtype glioblastoma
Source: J Neurooncol. 2026 Nov 10;180(2):43. doi: 10.1007/s11060-026-05855-2 (PMC13751740; doi:10.1007/s11060-026-05855-2)
Supplement: Supplementary file 8 [file 11060_2026_5855_MOESM7_ESM.docx]

**Supplementary Table 2.** DNA methylation classifier family assignments according to 14q status (n = 42)

| **Variable (Family)** | **14q loss (n = 17)** | **14q retained (n = 25)** | **P value** |
| --- | --- | --- | --- |
| Glioblastoma | 10 (58.8) | 14 (56.0) | 0.48 |
| Pediatric-type high-grade gliomas | 4 (23.5) | 5 (20.0) |  |
| Intermediate-grade IDH-wildtype gliomas | 1 (5.9) | 4 (16.0) |  |
| Low-grade glial/glioneuronal tumors | 2 (11.8) | 0 (0) |  |
| IDH glioma | 0 (0) | 1 (4.0) |  |
| Mesenchymal tumors | 0 (0) | 1 (4.0) |  |
