# Supplementary material for: Chromosome 14q loss is associated with adverse survival and a distinct transcriptional phenotype in IDH-wildtype glioblastoma
Source: J Neurooncol. 2026 Nov 10;180(2):43. doi: 10.1007/s11060-026-05855-2 (PMC13751740; doi:10.1007/s11060-026-05855-2)
Supplement: Supplementary file 9 [file 11060_2026_5855_MOESM8_ESM.docx]

**Supplementary Table 3. Sensitivity analyses of the multivariable association between chromosome 14q loss and overall survival in our cohort**

| **Model** | **14q loss HR (95% CI)** | **p** |
| --- | --- | --- |
| Prespecified model (Table 2) | 1.44 (1.02–2.01) | 0.036 |
| ***Treatment-related covariates and year of surgery*** | | |
| + bevacizumab | 1.41 (1.00–1.98) | 0.052 |
| + tumor-treating fields | 1.46 (1.04–2.05) | 0.031 |
| + year of surgery (per year) | 1.48 (1.05–2.08) | 0.025 |
| + bevacizumab, TTF, and year of surgery | 1.45 (1.02–2.04) | 0.036 |
| ***Canonical glioblastoma copy-number events (+7, −10, 9p loss, 13q loss, +19, +20)*** | | |
| + number of canonical events (0–6) | 1.44 (1.02–2.02) | 0.038 |

Each row is a separate Cox model for overall survival containing 14q status (loss vs. retained; hazard ratio shown), the prespecified covariates of Table 2 (age, preoperative KPS, total/subtotal resection, chemoradiotherapy, *TERT* promoter mutation, and *EGFR*, *PDGFRA*, *PTEN*, and *CDKN2A/B* alterations), and the covariate(s) named in the row.
