# Supplementary material for: Chromosome 14q loss is associated with adverse survival and a distinct transcriptional phenotype in IDH-wildtype glioblastoma
Source: J Neurooncol. 2026 Nov 10;180(2):43. doi: 10.1007/s11060-026-05855-2 (PMC13751740; doi:10.1007/s11060-026-05855-2)
Supplement: Supplementary file 10 [file 11060_2026_5855_MOESM9_ESM.docx]

**Supplementary Table 4. Expression of candidate chromosome 14q genes in relation to the 14q score and overall survival in our cohort (n = 215)**

| **Gene (cytoband)** | **Spearman ρ with 14q score (p)** | **Median log2 expression, 14q loss vs. retained (p)** | **Univariable OS HR per SD (95% CI; p; q)** | **Multivariable OS HR per SD (95% CI; p)** |
| --- | --- | --- | --- | --- |
| *NFKBIA* (14q13.2) | 0.23 (<0.001) | 5.36 vs. 5.81 (<0.001) | 0.85 (0.73–1.00; 0.046; q = 0.082) | 1.01 (0.85–1.20; 0.907) |
| *NDRG2* (14q11.2) | 0.27 (<0.001) | 7.51 vs. 8.04 (0.002) | 0.97 (0.83–1.12; 0.659; q = 0.659) | 1.01 (0.83–1.23; 0.922) |
| *MEG3* (14q32.2) | 0.19 (0.005) | 5.07 vs. 5.74 (0.004) | 0.76 (0.63–0.90; 0.002; q = 0.010) | 0.67 (0.55–0.81; <0.001) |
| *DICER1* (14q32.13) | 0.48 (<0.001) | 5.99 vs. 6.45 (<0.001) | 0.86 (0.75–1.00; 0.049; q = 0.082) | 0.84 (0.70–1.01; 0.057) |
| *TRAF3* (14q32.32) | 0.37 (<0.001) | 5.11 vs. 5.60 (<0.001) | 0.95 (0.83–1.08; 0.444; q = 0.555) | 0.95 (0.80–1.12; 0.547) |

Expression values are log2-transformed; hazard ratios (HR) are per standard deviation (SD) of log2 expression (higher expression vs. lower). q, Benjamini–Hochberg false discovery rate across the five genes. The multivariable model contains the gene, 14q status, and the prespecified covariates of Table 2 (age, preoperative KPS, total/subtotal resection, chemoradiotherapy, *TERT* promoter mutation, and *EGFR*, *PDGFRA*, *PTEN*, and *CDKN2A/B* alterations).
